# Parental guidance and problem solving in illustrated children’s books: the representation of the family as a pedagogical actor

**DOI:** 10.3389/fpsyg.2026.1915321

**Published:** 2026-09-30

**Authors:** Cigdem Cam Turkan

**Affiliations:** Department of Child Development, Faculty of Health Sciences, Iğdır University, Iğdır, Türkiye

**Keywords:** children’s literature, problem solving, children’s books, family, parental guidance

## Abstract

The preschool period is a critical time for developing emotional regulation and problem-solving skills. Children frequently encounter various stressors such as separation anxiety, peer bullying, and sibling rivalry, which require effective coping mechanisms. Parental support is crucial in developing children’s problem-solving skills. Children’s literature is an important resource in showing children the problems they may encounter and how to cope with them. This study aims to examine how the role of the family in children’s problem-solving processes is represented in illustrated children’s books and to identify the forms of support portrayed by family members within these narratives. The study was designed as a qualitative document analysis employing purposive criterion sampling to examine selected picturebooks as cultural products. Purposive sampling was used in the research. Ten illustrated books selected for this study were examined through content analysis. As a result of the analysis, the forms of parental participation in the process of developing children’s problem-solving skills in the books were categorized under the dimensions of “metaphorical concretization and cognitive guidance,” “metaphorical concretization and cognitive guidance,” “democratic participation and joint decision-making” and “play, role-playing, and creating a safe rehearsal space.” In the 10 works examined, parents participated in the problem solving process; They have “metaphorically concretized abstract concepts and Cognitive Guidance” “transformed social crises into safe rehearsals by gamifying them,” “exhibited participatory attitudes that encourage democratic participation in decision making processes,” and established an “unshakeable climate of trust through the emotional validation and reassurance strategy” they offered. The findings show that in children’s literature, the family is not merely a figurehead, but is positioned as an “external regulator” that helps children develop coping strategies for dealing with problems.

## Introduction

The level of attention and care given to a child should be consistent from the date of birth. The family providing care meets both the changing and emotional needs of the child. The greatest support in a child’s journey of individuation and personality development comes from the family. Developing the basic architecture of the brain is not solely determined by genes; many different experiences children offer contribute to this development (Siegel and Bryson, 2015). According to Yapıcı (2010), the family is where children’s education is best realized. The most important values for the existence of societies are passed on to new generations. The family is the first model to follow in finding solutions to problems. The family uses various tools in child education, one of which is children’s literature. Children’s literature provides a kind of education and socialization by conveying society’s goals, fears, ratios, and shares (Landt, 2006; Louie, 2006). Alver (2005) stated that children discover the literature of life. Children’s literature, particularly illustrated children’s books for early childhood, serves as a source for children to gain their first experiences with entertaining literature (Feyman and Tuğrul, 2006). Children’s literature should support children, communicate with them, instill a love of reading, and guide them towards free and critical thinking (Dilidüzgün, 2018). Children, whose perspectives on life differ from those portrayed by the characters, identify with the heroes and discover their characteristics. The world in which the author emerges is where the stars of the countries are. Furthermore, by witnessing different lives, children can understand society and people (Zivtci, 2007). For this reason, the characteristics of families, their transformations, and the way they address problems are quite important for readers in the structures of children’s literature. To expand the solution process and initiation strategies in these relationships, children’s literature utilizes children’s books to teach families these kinds of behaviors. Illustrated books are books that provide visual and verbal experiences, consisting of large collections of pictures (Owens and Nowell, 2001). Picture books play a significant role in the lives of preschool children. The benefits of picture books for children are limitless. For example, they serve as models for narrative language and provide aesthetic experiences. Through books, children develop a sense of art, their language development is supported, and they learn about their environment. Since illustrations constitute the majority of a book, picture books provide children with information about aesthetic skills, emotions, and many themes (Harper, 2001). There are also picture storybooks that address events and problems children may encounter in their lives (the birth of a new sibling, moving to a new place, the first day of school, etc.) and are used to help them cope with these events (Alexander et al., 2001). Preschool children can make sense of emotions they feel but cannot easily express, emotions they are trying to explore, through picture books. Children find reflections of themselves in books and interact with the characters, the scene, the time, the events, and their consequences, thus becoming immersed in the narrative (Ewans, 2013). According to Sever (2013), the character’s attachment to life, their determination to solve the problems they face, the quality of the methods they follow to achieve their goals, and the reflection of these in their words and actions, motivate children to behave like the characters in the book. Turan (2010) stated that children are guided in coping with problems and difficulties, and in solving emotional and social problems. While children’s literature resources are not the primary tools for helping children, various literary works can be used as effective tools for children in the context of problem-solving. From this perspective, children can understand that they are not alone in facing problems. Problem-solving skills are the ability to cope with uncertainties and to respond appropriately and flexibly to a problem situation despite internal and external factors (Metwally and Akyol, 2018; Dow and Mayer, 2004). It is seen that establishing and maintaining these skills in children is critically important for them to effectively use coping strategies. Through activities included in the educational curriculum during this period, children can be enabled to recognize, experience, and evaluate these strategies (Uysal Bayrak et al., 2018). Children who can respond appropriately to problems from the early years of life will experience less anxiety and make healthier decisions when faced with problems in adulthood. It has been observed that interpersonal relationships are important in coping with problems and emotion regulation skills, and that an individual’s emotion regulation skills are directly related to their social competence (Barthel et al., 2018; Marroquín et al., 2017). According to Lazarus and Folkman (1988), coping style plays a significant role in the emergence of emotional responses as a mediator in individuals’ evaluations of events they encounter in their lives. Many researchers distinguish between active coping (positive), which involves confronting the problem, and coping (negative), which involves avoiding confrontation (Billings and Moos, 1981; Roth and Cohen, 1986). A distinction can be made between functional and dysfunctional coping styles. Functional coping strategies involve actively seeking support to cope with the problem, engaging in concrete solution-oriented behaviors, or exploring possible solutions; while dysfunctional strategies may involve ignoring negative emotions and not exploring possible solutions to the problem (Kayri and Boysan, 2007). Children have been found to use the following positive strategies to cope with their anxiety and fears: diverting attention to another task, trying to solve the problem, seeking adult support, seeking relaxation/calming down, thinking positively, expressing their feelings, and expressing themselves humorously. Negative strategies include doing nothing/not knowing what to do, crying/shouting/throwing tantrums, anger, ignoring the problem, blaming others, blaming themselves, hiding their feelings, and exhibiting aggressive behavior (Chalmers et al., 2011). According to Hansen and Zambo (2005), picture books should be used by child development specialists and educators as a strategy to improve children’s cognitive, physical, and socio-emotional development, as well as their coping skills with negative experiences and emotions. Children’s literature is a wonderful material that teaches about the relationship between parents and children, and between siblings and peer groups (Stegmaier, 1974). The concept of family and how family is portrayed to children in children’s books is an issue that needs to be examined. The family institution, which has maintained its importance throughout history, has become even more significant in today’s conditions due to societal changes. Ensuring social continuity, transmitting values among individuals, serving as a role model in problem-solving, and playing a crucial role in children’s education necessitate the family’s important position in every era (Karadağ, 2023).

Research on picture books emphasizes that meaning is produced not only through written text but also as a result of the interaction between text and visual elements (Nikolajeva and Scott, 2013). In this context, children, while understanding the characters, plot, and verbal narrative in the story, also interpret the emotional expressions, body language, spatial relationships, and symbolic signs presented in the visuals. Thus, picture books enable children to make sense of complex social and emotional experiences in a way that is appropriate to their developmental level. In this respect, the visuals in picture books are not only elements that support the text; they also have the ability to create their own unique system of meaning. According to Kress and van Leeuwen (1996), visuals have a unique grammar that conveys social relationships, power dynamics, emotional states, and cultural values. Similarly, Serafini (2014) states that the multi-layered meaning structures of picture books can only be understood by considering the text, visuals, color, perspective, composition, and symbolic elements together.

The role of the family in child development is crucial, particularly in the acquisition of emotional regulation and problem-solving skills. Morris et al. (2007) state that parents are fundamental socializers in the processes of recognizing, expressing, and regulating children’s emotions. Parents help children cope with the difficulties they encounter by modeling, guiding, offering emotional validation, and establishing supportive communication. These processes support not only children’s emotional development but also their problem-solving skills. These supportive processes that children experience within the family are frequently represented in children’s literature. Harper (2016) states that illustrated children’s books are powerful tools that support social–emotional learning and offer children the opportunity to observe different coping strategies in the face of problems experienced by characters. By identifying with book characters, children can make sense of their emotions, discover ways to resolve social conflicts, and develop alternative problem-solving strategies. Although there are various studies in the literature on family representations, values education, social–emotional learning, and problem-solving skills in children’s literature, studies examining the pedagogical functions of family members in children’s problem-solving processes within the textual and visual narrative structures of picture books are limited. While the vast majority of existing studies focus on the presence of family contact or how problem-solving skills are addressed in the books, they do not comprehensively address the pedagogical strategies through which families guide children in their problem-solving processes and how this guidance is represented through visual and verbal indicators.

It is stated that children can acquire many pieces of information and skills through picture books for children. Similarly, it is clear that children tend to use functional or dysfunctional coping strategies when faced with any negative situation. Showing children how their family behaves in problem-solving in books for children is an important issue to focus on. The aim of this research is to identify and examine how family members cope with the problems they encounter in picture books for children, what solutions they produce, and how these solutions affect children.

This research is significant because it reveals that children’s literature is not merely a means of entertainment, but also a guide that plays a role in children’s development and in acquiring problem-solving skills. It is expected that the analysis of family problem-solving attitudes in children’s books will contribute to future children’s books and serve as a resource for scientific studies in this field. This research aims to examine the roles families play in the problem-solving processes of children facing individual, social, and emotional problems in illustrated children’s books published between 2010 and 2025. By analyzing the guidance functions of parents in the problem-solving process under thematic categories and evaluating these representations within a visual-textual context, the study aims to provide a theoretical and methodological contribution to the existing literature on the relationship between family and problem-solving in illustrated children’s books. Existing studies have examined family representations, values education, or problem-solving skills in illustrated children’s books separately. However, studies investigating how family members guide child characters in their problem-solving processes, through which pedagogical strategies (emotional coaching, metaphorical concretization, gamification, democratic participation, etc). Although previous studies have examined the educational value of picturebooks, their contribution to children’s socio-emotional development, and the role of families in children’s learning, most of this research has primarily focused on developmental outcomes, educational interventions, or the pedagogical use of picturebooks. Comparatively less attention has been given to how family guidance and parental problem-solving are represented within the narratives and visual-textual structures of picturebooks themselves. The present study addresses this analytical focus by examining how parents are portrayed as pedagogical actors during children’s problem-solving processes across the selected picturebooks, rather than evaluating the effectiveness of these parenting practices. Around which themes are the problems encountered by child characters in illustrated children’s books structured?

What pedagogical roles do family members assume in solving these problems?

How are these pedagogical roles represented through texts?

## Method

### Research model

This study was designed as a qualitative document analysis. Document analysis is a systematic qualitative research method used to examine, interpret, and analyze written and visual materials related to a particular phenomenon (Bowen, 2009). Within the scope of this study, illustrated children’s books were treated as cultural and pedagogical documents reflecting family–child interactions and problem solving practices. The documents were analyzed using qualitative content analysis, which enabled the systematic identification, coding, categorization, and interpretation of recurring themes and patterns related to how family members respond to problems, the solution strategies they employ, and the potential educational implications of these strategies for children. Accordingly, document analysis constituted the overall research design, whereas qualitative content analysis was employed as the analytical procedure. This methodological framework ensured a systematic and rigorous examination of the selected picturebooks.

### The universe and sample of the study

The research universe consists of picture books from different publishers and authors. The sample was selected using the “criterion sampling” method, a type of “purposive sampling,” in line with the study’s objective. The data source consisted of picturebooks published between 2010 and 2025. Criterion sampling, a type of purposive sampling, was employed to select information-rich cases relevant to the research purpose (Patton, 2015). In this sense, the purposive sampling method is useful in explaining many situations, phenomena, and events. With criterion sampling, a type of purposive sampling, it is possible to study situations that meet a set of criteria predetermined by the researcher (Yıldırım and Şimşek, 2013). The search for eligible books was conducted between January and March 2025 using major Turkish online booksellers (e.g., Kitapyurdu, D&R, İdefix), publishers’ catalogues, and online bookstore databases. Search terms included “picturebook,” “preschool,” “family,” “mother,” “father,” “problem solving,” “emotion,” and “children’s book.” Only Turkish-language editions published between 2010 and 2025 were considered. The initial search identified 76 titles. After screening titles and book descriptions, 24 books were retained for full-text examination. Fourteen books were excluded because they did not involve family participation, lacked a central problem situation, targeted older readers, or were informational books. The remaining 10 books satisfied all inclusion criteria and were included in the document analysis. The remaining 10 books satisfied all inclusion criteria and were included in the document analysis. The publication period of 2010–2025 was chosen because contemporary picturebooks increasingly emphasize social–emotional learning, emotional literacy, family relationships, and child-centered problem-solving approaches. Examining books published during this period enabled the study to capture current pedagogical trends and family representations. The inclusion criteria were intentionally designed to identify picturebooks portraying constructive forms of parental guidance during children’s problem-solving processes. Accordingly, the present study should not be interpreted as representing the full spectrum of family portrayals in contemporary children’s literature, such as absent, ineffective, controlling, harmful, or ambivalent parenting. Rather, the selected corpus constitutes a purposive sample of information-rich cases illustrating positive family guidance within children’s narratives. Therefore, the findings are limited to these selected representations and should not be generalized to all picturebooks or all family portrayals.

The inclusion criteria were:

* being a picturebook intended for children aged 3–6,

* being published between 2010 and 2025,

* containing a clearly identifiable problem situation,

* including at least one family member participating in the problem-solving process,

* containing both visual and verbal narratives.

The exclusion criteria were:

* informational books without a narrative structure,

* books without family involvement,

* books targeting readers older than 6 years,

* books in which the central conflict was not resolved within the narrative.

Ten picturebooks meeting these criteria were selected and analyzed. The bibliographic information of the boks examined in the study is given in Table 1.

**Table 1:** Bibliographic information of the books forming the data source.

Book Title/publisher name	Book code	Publication year	Domestic (D)/translation (T)	Character type
Will You Still Love Me?/Meav	1.	2021	T	Animal
Democracy at Home/Eğiten Kitap	2.	2018	D	Animal
Unshared Pens/Cezve Children	3.	2021	D	Human
The Badger Who Couldn’t Sleep/İş Kültür Publishing	4.	2021	T	Animal
Tali Copes with Bullying/Nesil Children’s Publications	5.	2018	D	Animal
Being a Baby Octopus is Very Hard/YKY Publishing	6.	2023	D	Animal
The Kissing Hand/Butik Publishing	7.	2013	T	Animal
İnci Does not Go with Strangers/İş Kültür Publishing	8.	2018	T	Human
Which of Us Do You Love Most?/Uçanbalık Publishing	9.	2024	T	Animal
Bad Words Smell Bad/Nar Publishing	10.	2020	T	Animal

### Data collection tools

Data were collected using a “Book Information Form,” prepared by research based on a literature review and finalized in accordance with expert opinions. The Book Information Form was used to determine the information of the illustrated children’s books used in the research. Each book was assigned a number in the Book Information Form. Information such as the year of publication, publisher, whether the book is a translation or not, and whether the protagonist is human, animal, or object was recorded on this form. The parents’ approach to problem-solving was also recorded on the form. After reading each book to a satisfactory extent, the researcher recorded the data in a checklist. After obtaining expert opinions, the researcher completed the checklists for all the books. In this research, the ways in which parents support the problem-solving skills of the protagonist were examined and recorded in the data collection instrument.

### Data analysis

The picturebooks were analysed as multimodal texts in which meaning was constructed through the interaction of verbal and visual modes. Accordingly, coding was based on the integrated interpretation of textual and visual information rather than on either mode in isolation. During the analysis, visual features such as facial expressions, gaze, body posture, gestures, spatial positioning, and contextual visual cues were examined together with the accompanying text to identify how parental guidance and children’s problem-solving processes were represented. Particular attention was also paid to the relationship between verbal and visual information to determine whether the illustrations reinforced, complemented, or extended the narrative meaning.

Content analysis was used in the analysis of the data. “Content analysis is a technique that makes inferences about the objective and systematic identification of certain characteristics of a message. Researcher identify and analyze the existence, meanings, and relationships of these words and concepts, and make inferences about the message in the texts (Büyüköztürk et al., 2019).”

In the first stage, all books were read several times and coded according to the problem type, family involvement, coping strategy, and pedagogical support provided by family members.

In the second stage, codes were grouped into broader themes representing family problem solving approaches. Through an inductive analytical process, three overarching themes emerged:

1. Emotional coaching.
2. Cognitive guidance.
3. Modeling and participatory problem solving.

The final interpretations were developed by examining how textual and visual elements jointly contributed to the construction of these themes. After the data were organized according to this list, the findings were discussed according to these criteria and interpreted by the researcher. For the reliability of the research, all illustrated children’s books were independently examined by preschool and Turkish language teachers, and a reliability rate of 90% was achieved using Miles and Huberman's (1994) formula (Reliability = agreement/agreement + disagreement). In qualitative studies, a desired level of reliability is achieved when the agreement between expert and researcher evaluations is 70% or higher.

### Unit of analysis

The primary unit of analysis in this study was each problem-solving episode involving a child character and at least one family member. A problem-solving episode was defined as a coherent narrative sequence in which (a) a problem emerged, (b) a family member provided guidance or support, and (c) the child attempted or completed the resolution of the problem. Each episode was analysed through both verbal (textual) and visual (illustrative) representations. Individual illustrations and textual excerpts were not coded separately but were interpreted together as complementary components of the same narrative event.

### Ethical approval of the study

This study complied with all the rules specified in the “Guidelines for Scientific Research and Publication Ethics in Higher Education Institutions”. None of the actions listed under the second section of the guidelines, titled “Actions Contrary to Scientific Research and Publication Ethics,” were carried out. To enhance the credibility of the findings, coding procedures were independently reviewed by experts in early childhood education and children’s literature. Intercoder agreement was calculated using the Miles and Huberman (1994) formula and reached 90%, indicating a high level of consistency. Disagreements were discussed until consensus was achieved.

## Findings

This section will first present the findings of the 10 illustrated children’s books selected as the sample in the research. The findings presented below are based on the integrated analysis of both verbal and visual representations. All interpretations were derived from the combined examination of textual and illustrative evidence within each problem-solving episode.

Book 1: In the book “Will You Still Love Me?”, the problem of the child’s anxiety about not being loved is addressed at an individual level. This problem is frequently encountered as a reflection of attachment-based insecurities and conditional acceptance perceptions in early childhood. The solution approach offered in the work is educational and realistic. The parent is seen to respond to the child’s emotional needs with an emphasis on unconditional love. Family members are seen to play a supportive role in the problem-solving process. The parent figure is seen to provide a model that gives the child confidence and exhibits an explanatory attitude. Family members who patiently listen to the child’s anxieties are portrayed in the narrative as providing emotional support. This representation emphasizes the value of family-based emotional reassurance within the story, although its interpretation should be understood as reflecting the narrative rather than prescribing a universally applicable parenting practice.

In Figure 1, the little bear cub asks questions to find out what conditions his mother’s love for him depends on: “His mother opened the sewing box. Martin restlessly circled it. ‘What’s wrong, son?’ asked his mother, searching for the right color thread. ‘Did I do something wrong? Do you still love me?’ ‘Of course I do!’”

**Figure 1:**
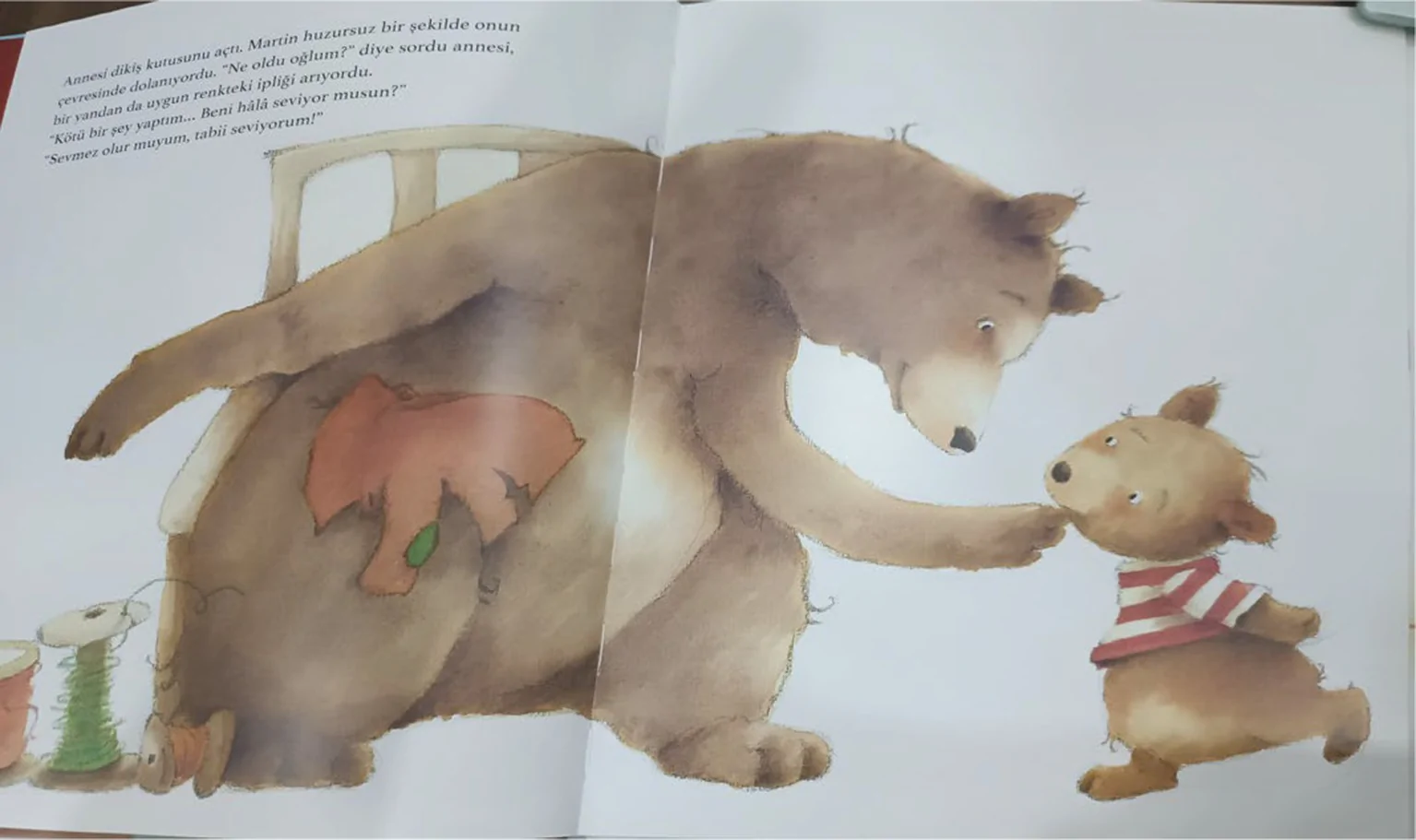
“Will You Still Love Me?” book (Leblanc, 2018). “Est-ce que tu m’aimeras encore?” book (Reproduced with permission from Leblanc, 2012). © 2012 minedition france, Est-ce que tu m’aimeras encore?, texte de Catherine Leblanc et illustrations d’Ève Tharlet.

The central problem presented in the book revolves around Little Bear Martin’s anxiety about not being loved, which develops after a physical mistake (tearing his coat). The honest and calm attitude of the mother character during this process is a decisive factor in alleviating the child’s anxiety. Instead of ignoring Martin’s mistakes, the mother responds with a realistic approach, saying, “I scold him, I get upset, but I still love him.” Through Martin, the narrative promotes the value of unconditional parental love as an educational message. From an analytical perspective, this representation may support children’s sense of emotional security; however, the present study interprets this as a narrative value rather than empirical evidence of the effectiveness of a specific parenting approach (see Figure 2).

**Figure 2:**
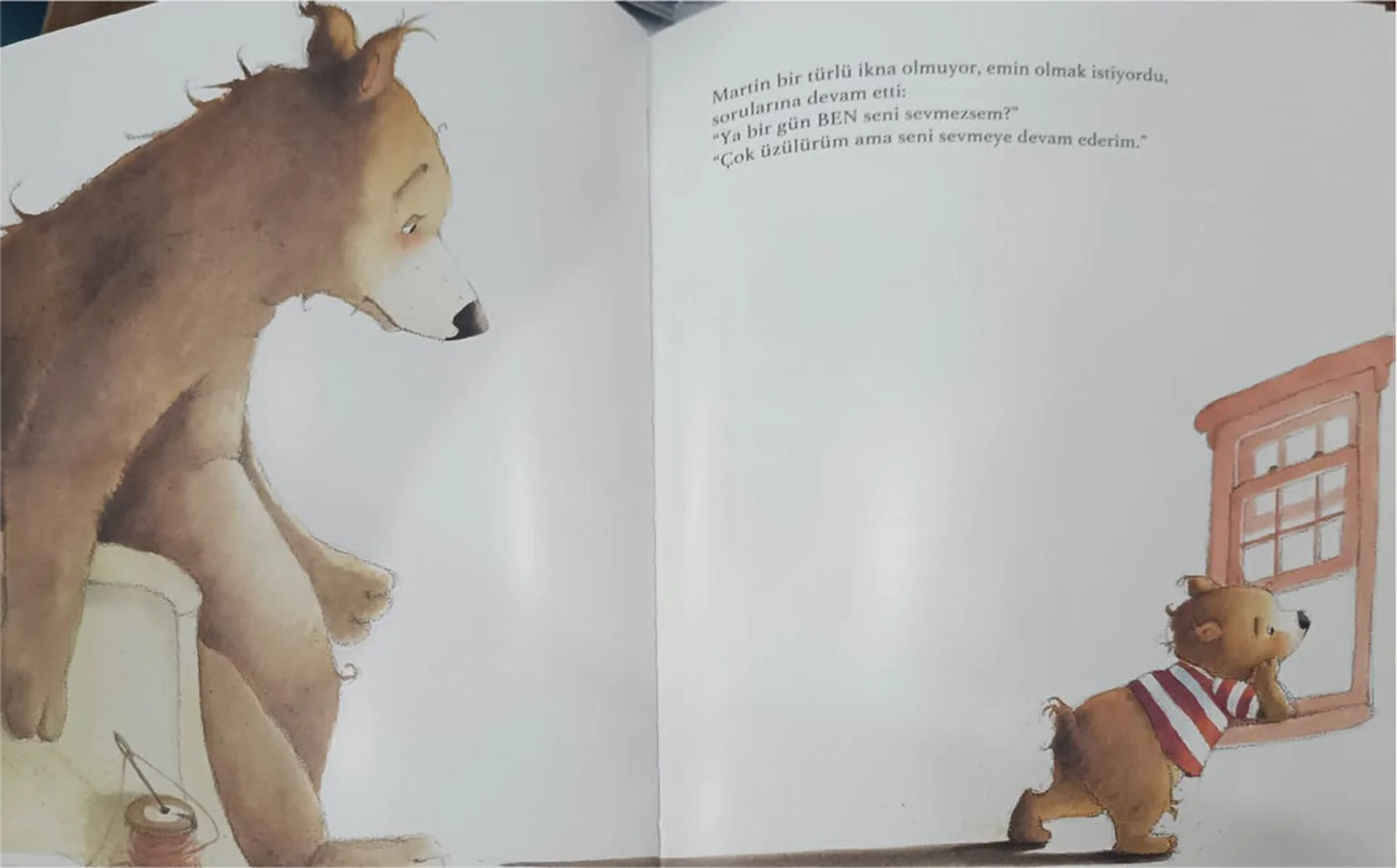
“Will You Still Love Me?” book (Leblanc, 2018). Est-ce que tu m’aimeras encore??” (Reproduced with permission from Leblanc, 2012). © 2012 minedition france, Est-ce que tu m’aimeras encore?, texte de Catherine Leblanc et illustrations d’Ève Tharlet.

Book 2: In the book titled “Democracy at Home” the problem of limited budget and resource sharing is addressed at the family level. This problem, directly related to social skills such as justice, equality, and resource distribution that children may encounter at an early age, is frequently encountered. The solution approach offered in the work is based on a logical and analytical foundation. The solution is concretized, particularly through the use of democratic decision-making processes such as voting and sharing. Family members are seen to assume a guiding and instructive role in the problem-solving process. The parent figure is presented as a model that suggests democratic solutions and guides children in implementing these solutions. In this context, the parents in the narrative are presented as modelling democratic participation within the family. The narrative promotes shared decision-making as a constructive family value, although the suitability of this approach may vary depending on children’s developmental characteristics and the family context.

In Figure 3, the parents present the idea of fulfilling their children’s wishes and resorting to democracy when no consensus can be reached. “So, what now?” *said the father fox, sitting despairingly.* “Why don’t we have a vote?” *asked the mother fox.* “Whoever’s idea gets the most support will receive the gift. That way we can fairly determine which gift we get.”

**Figure 3:**
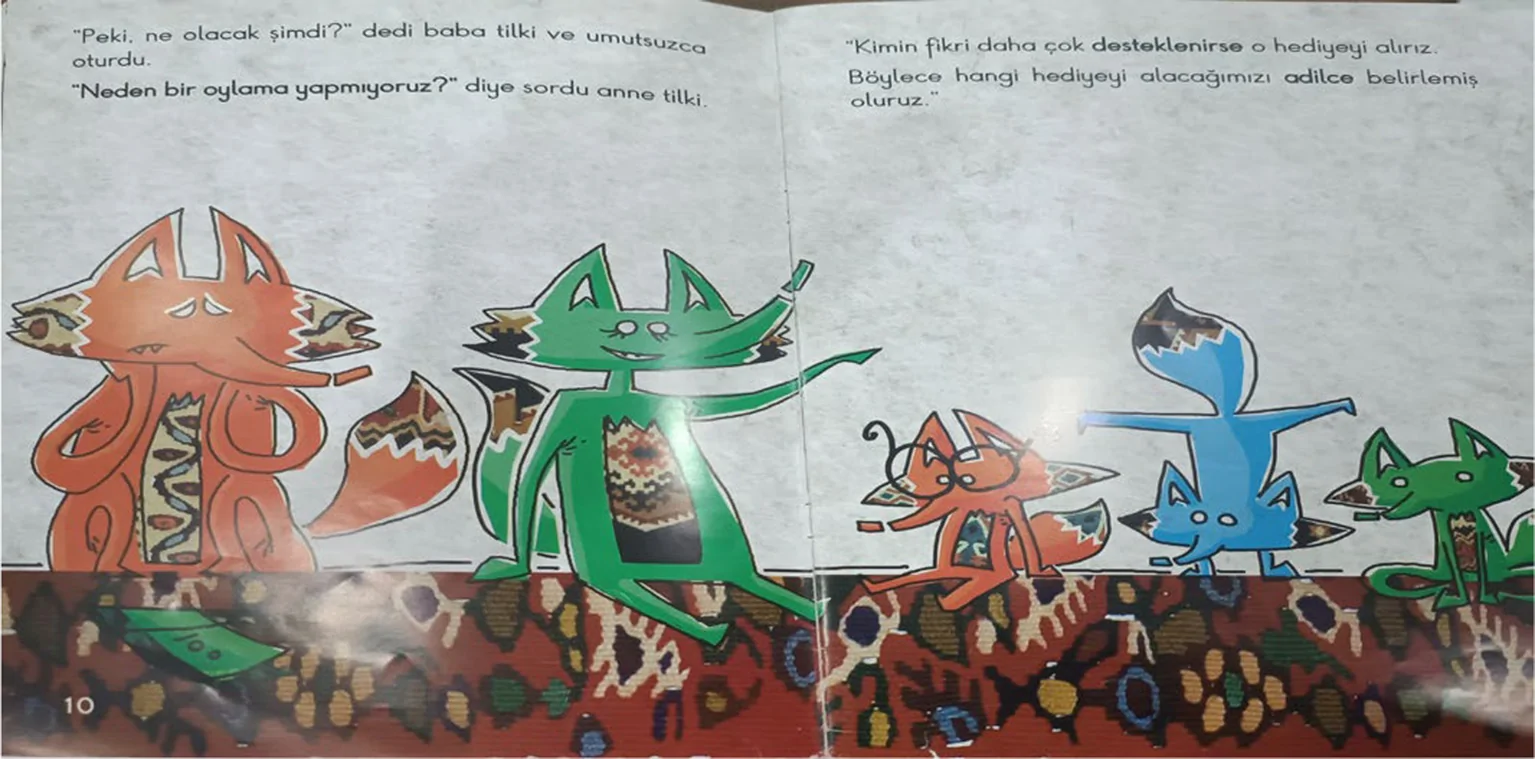
“Democracy at Home” book (Reproduced with permission from Savaş, 2018)

The central problem presented in the book is how a limited family budget is shared despite the differing demands of family members. Instead of exhibiting an authoritarian attitude, the characters Mother and Father Fox involve their children in voting and logical argumentation processes, illustrates a narrative representation of democratic participation in family decision-making. Rather than suggesting that this approach is universally optimal, the story highlights democratic dialogue as one possible educational value.” The book offers readers valuable guidance on how families can transform moments of crisis into educational opportunities.

Book 3: In the book “Unshared Pens” the problem of ownership perception and conflict between siblings is addressed within the family context. Problems related to ownership, boundaries, and sharing are frequently encountered as a natural part of children’s development of basic social–emotional skills. The solution approach offered in this work is based on emotional sharing. Specifically, the conflict is resolved through siblings empathizing with each other and apologizing. Family members play a supportive role in the problem-solving process. The parent figure acts as a mediator, encouraging children to establish healthy communication and providing a model that helps children name their emotions. In this context, the parents in the narrative can be considered role models demonstrating that family conflicts can be resolved through constructive and emotionally aware approaches (see Figure 4).

**Figure 4:**
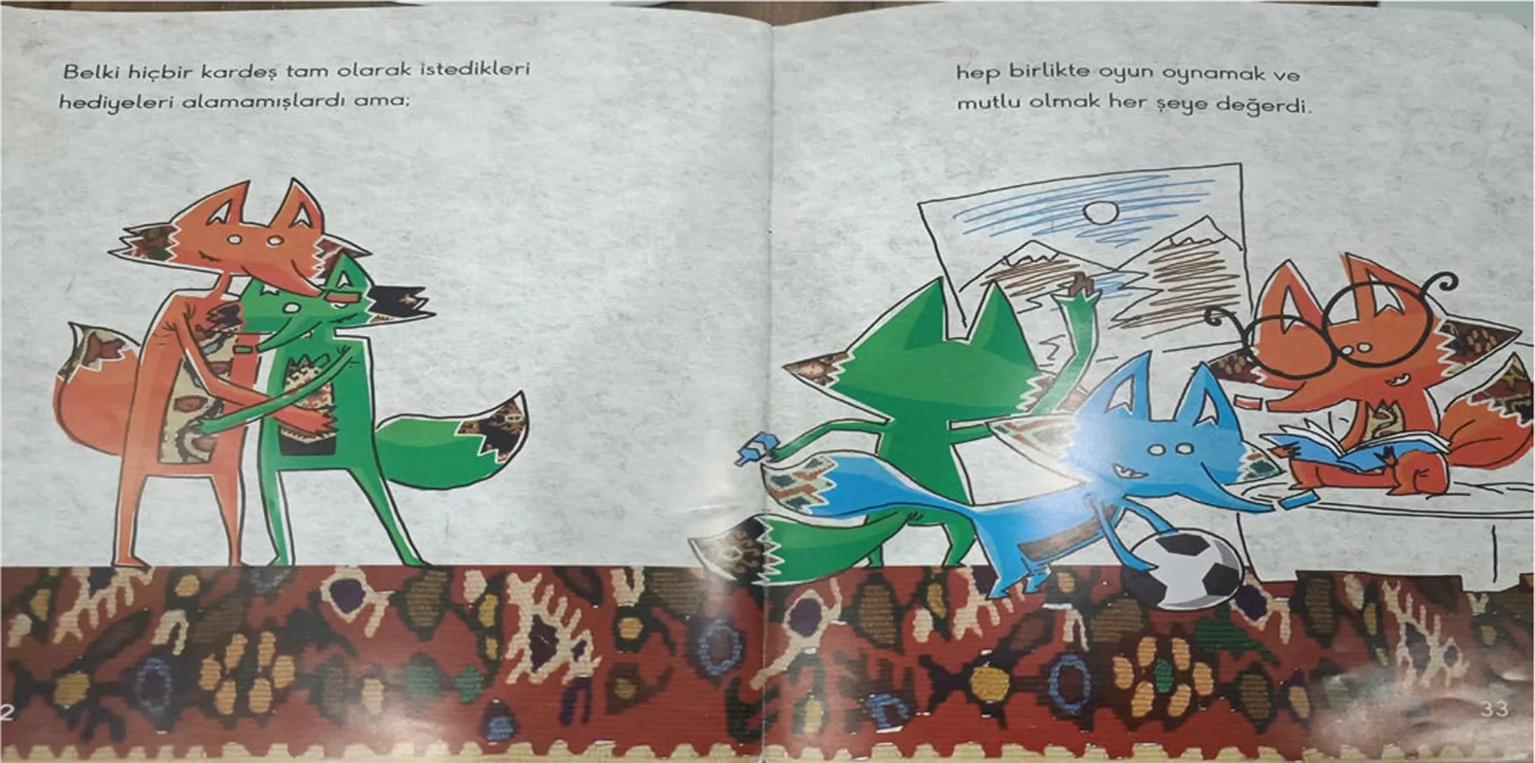
“Democracy at Home” book (Reproduced with permission from Savaş, 2018).

In Figure 5, Anne Zehra prompts Alperen to reflect on what he did. “His mother calmly turned to Alperen and said, ‘We don’t treat each other like that, do we, Alperen?’ Then, turning to Zehra, she added, ‘I think you were hurt and very upset because your brother treated you harshly.’”

**Figure 5:**
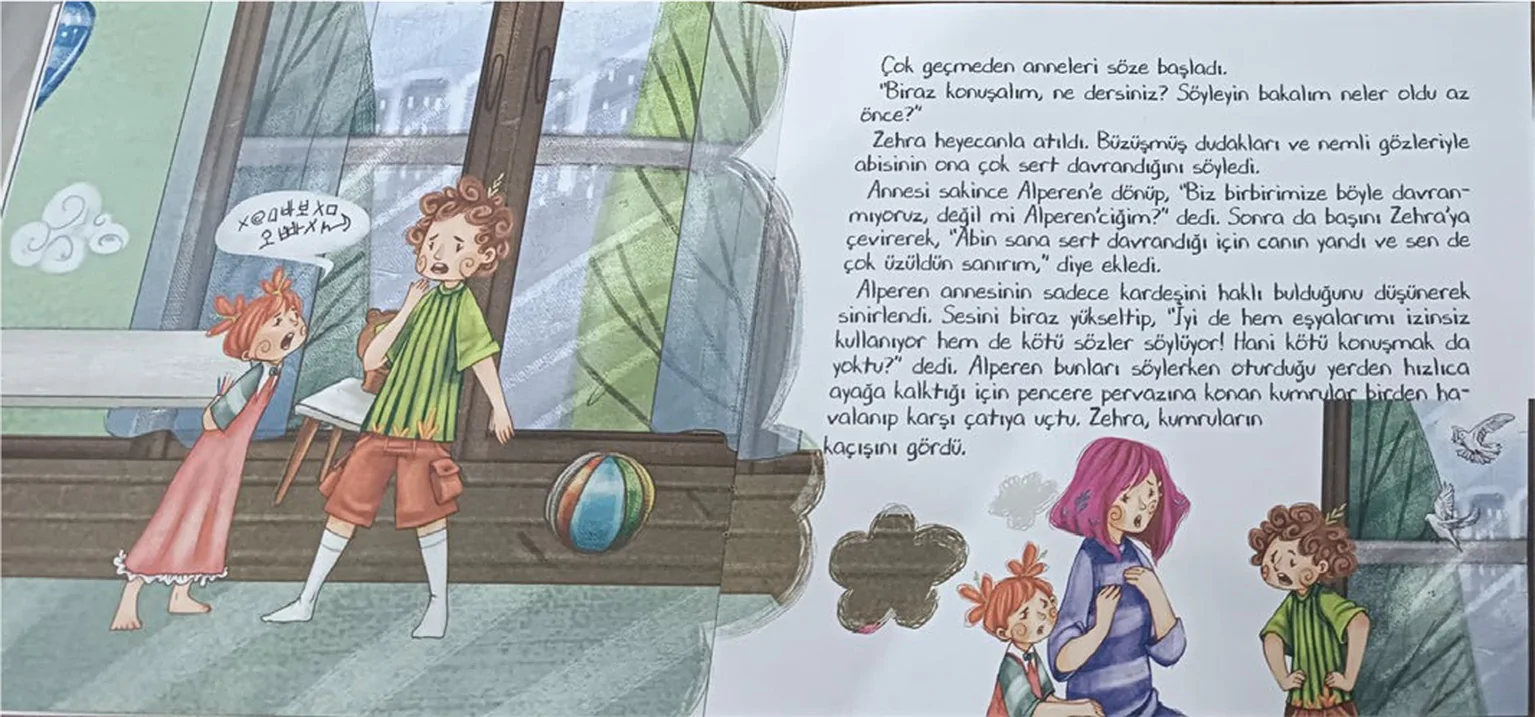
“Unshared Pens” Book (Reproduced with permission from (İnan and Kardaş, 2023).

The book successfully exemplifies the mother’s mediating role in managing sibling conflicts. The central problem in the book is the unauthorized use of belongings and the resulting faulty communication patterns. The solution is achieved through the parent’s use of concrete analogies and highlighting emotions. The mother character assumes the role of a “facilitator/mediator,” enabling children to recognize their mistakes. The use of the “key” metaphor represents one narrative strategy for explaining the concept of asking permission in a concrete manner. Although this representation may facilitate children’s understanding of property boundaries, its educational effectiveness would depend on children’s developmental characteristics and the broader educational context.

Book 4: “The Badger Who Couldn’t Sleep” is structured around a problem at the individual/internal level, addressing a child’s resistance to self-care skills and daily routines. Problems in boundary setting and self-regulation processes are frequently encountered in early childhood. The solution approach in the work is based on a realistic foundation. It is understood that the goal is to regulate behavior, particularly through the consistent and stable completion of routines by parents. In this respect, the work offers a functional framework for teaching children daily living skills and supporting the development of self-discipline. The family plays a guiding and instructive role in the problem-solving process. The parental figure guides the child in internalizing routines by exhibiting a patient, consistent, and boundary-setting attitude. In this respect, the parents in the narrative is portrayed as highlighting the potential value of structured family routines within the narrative. This interpretation reflects the educational message of the story rather than a universal recommendation for parenting practice (see Table 2).

**Table 2:** Operational definitions and coding framework used in the analysis.

Concept	Operational definition	Coding indicators
Family	Parents or other family members (e.g., mother, father, siblings, grandparents) who actively participate in the child’s problem-solving process within the narrative.	Presence of a family member providing support, guidance, or intervention.
Problem	Any cognitive, emotional, social, or practical challenge encountered by the child character that requires resolution.	Conflict, obstacle, fear, misunderstanding, interpersonal disagreement, or task difficulty.
Problem-solving	The sequence of actions through which the child, with or without family support, attempts to resolve the identified problem.	Identification of the problem, guidance provided, child response, and outcome.
Parental guidance	Any verbal or non-verbal support provided by a family member to facilitate the child’s understanding, coping, or resolution of a problem.	Advice, explanations, questioning, encouragement, comforting, demonstration, or shared decision-making.
Emotional coaching	Guidance that helps the child recognize, understand, express, or regulate emotions.	Validating feelings, comforting, empathy, discussing emotions, emotional reassurance.
Cognitive guidance	Guidance intended to stimulate thinking, reasoning, reflection, or independent problem-solving.	Asking guiding questions, providing clues, explanations, prompting reflection, encouraging alternative solutions.
Modeling	Demonstration of appropriate behaviors or strategies that the child can observe and imitate.	Parent demonstrates desired behavior before or during problem-solving.
Democratic participation	Family involvement that encourages the child to express opinions, participate in decision-making, and collaborate in finding solutions.	Shared decision-making, discussing alternatives together, respecting the child’s ideas.
Safe rehearsal	Creating opportunities for the child to practice new skills or behaviors within a supportive and low-risk environment.	Role-playing, guided practice, pretend play, supervised attempts before real-life application.
External regulator	Temporary regulation of the child’s behavior, emotions, or actions by a family member until the child can self-regulate.	Parent sets limits, provides reminders, organizes actions, or scaffolds the child’s behavior.

In Picture 7, Father Badger supports the little badger character in fulfilling his responsibilities. “Little Badger tidied his room, ate his meal, and brushed his teeth with your help. So, what’s next? Little Badger’s father said, ‘It’s time to read a book.’ Little Badger happily said, ‘Yay! Let’s start right away!’ Father asked Little Badger, ‘Which book should you read to him? Will you choose?’”

The main problem in the book is that we see Little Badger using the excuse of “tiredness” to avoid responsibilities. We see that this situation develops a zone of patience in the parent–child relationship. We see that the parents exhibit a calm and firm attitude despite the child’s resistance. In “The Badger Who Couldn’t Sleep,” we see that parental guidance, through the book, moves from a directive role to a collaborative one. The badger father does not just dictate routines; he invites the child into the decision-making process. A striking and unique feature of the book is that, thanks to its “interactive book” feature, it makes the reader a part of the solution (see Figure 6).

**Figure 6:**
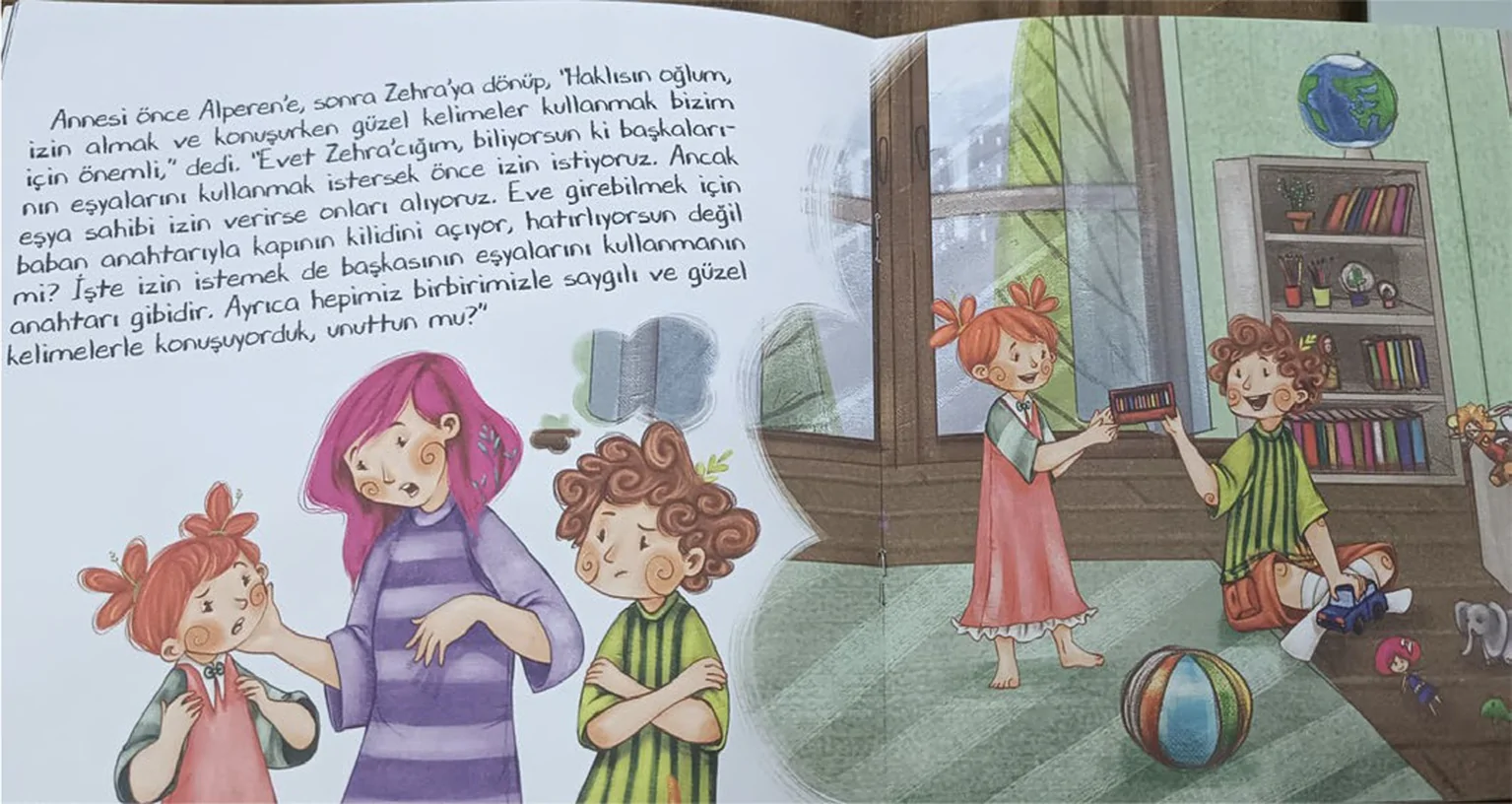
“Unshared Pens” book (Reproduced with permission from İnan and Kardaş, 2023).

Book 5: The book, “Tali Copes with Bullying” is structured around peer bullying in the social sphere and the psychological resilience developed against it. It is crucial for children to develop coping skills against these negative interactions they may encounter in their social environment. The solution approach presented in the narrative emphasizes reducing conflict through an avoidance-based strategy, particularly by temporarily ignoring bullying behaviors. Within the story, this strategy is portrayed as one possible coping response rather than the only or universally appropriate solution. Depending on the severity, persistence, and context of bullying, alternative responses, including seeking support from trusted adults or teachers, may also be appropriate. The focus is on reducing the impact of conflict through a strategy of ignoring the parental bullying behavior. In this context, through the parent, it is shown that alternative coping mechanisms are possible instead of directly engaging in conflict in every situation. The family’s role in the problem-solving process is presented as guidance and support. The parent teaches coping strategies to the child through play-based methods (using puppets). In this respect, the book provides exemplary pedagogical content aimed at increasing children’s social–emotional resilience (see Figure 7).

**Figure 7:**
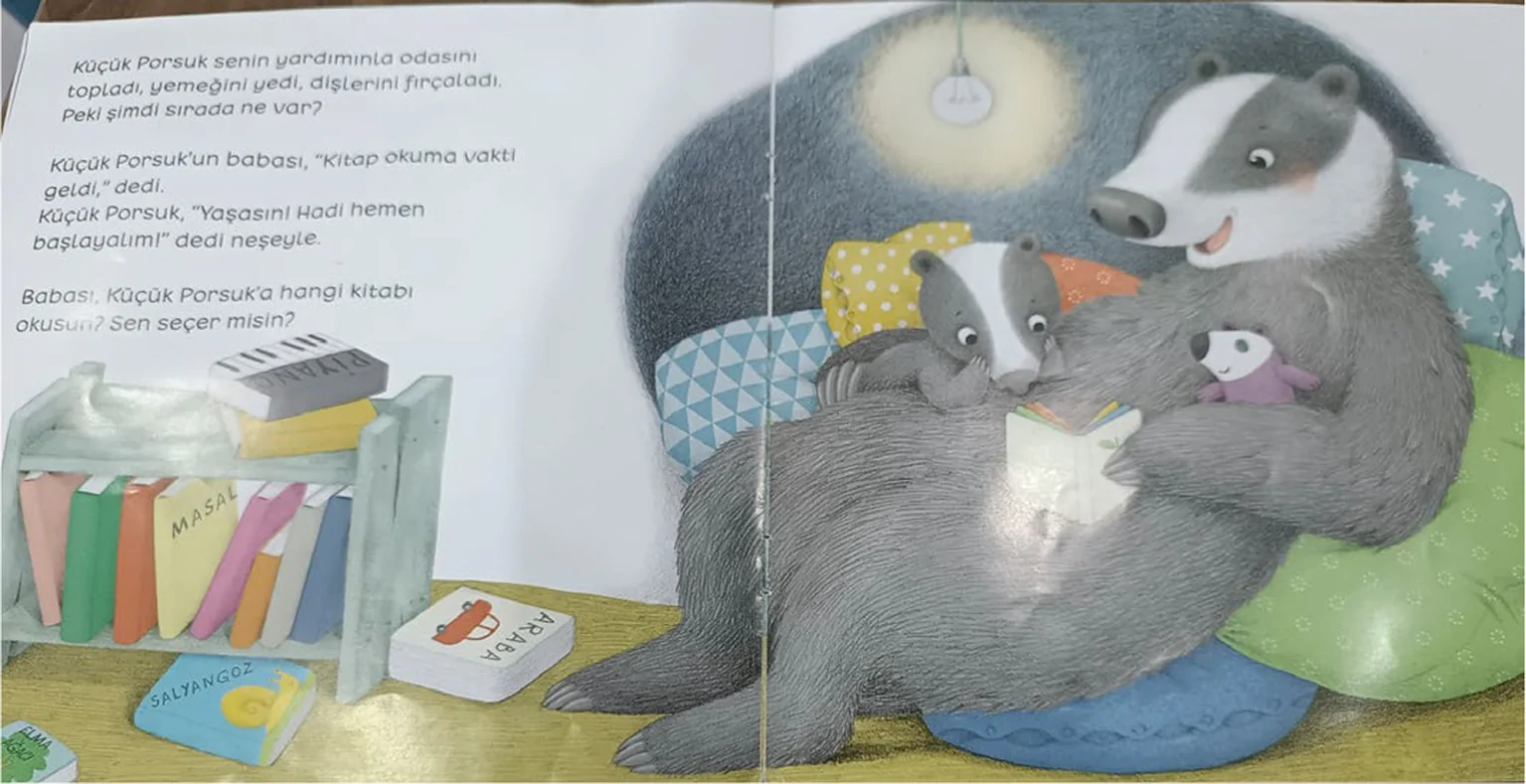
“The Badger who could not Sleep” book (Scarpa, 2020). “Ich bin gar nicht müde!” book (Reproduced with permission from Von Kitzing, 2019).

In Figure 8, Tali’s mother is supporting her in coping with the bullying she experienced at school. “Her mother said, ‘You’re right, darling, you’re in a difficult situation,’ and waited for her to calm down. After listening to Tali, she brought two puppets. One puppet became Tali, and the other became Tintin. Tali loved this game. While watching the puppets, she also learned what to do if she was bullied again.”

**Figure 8:**
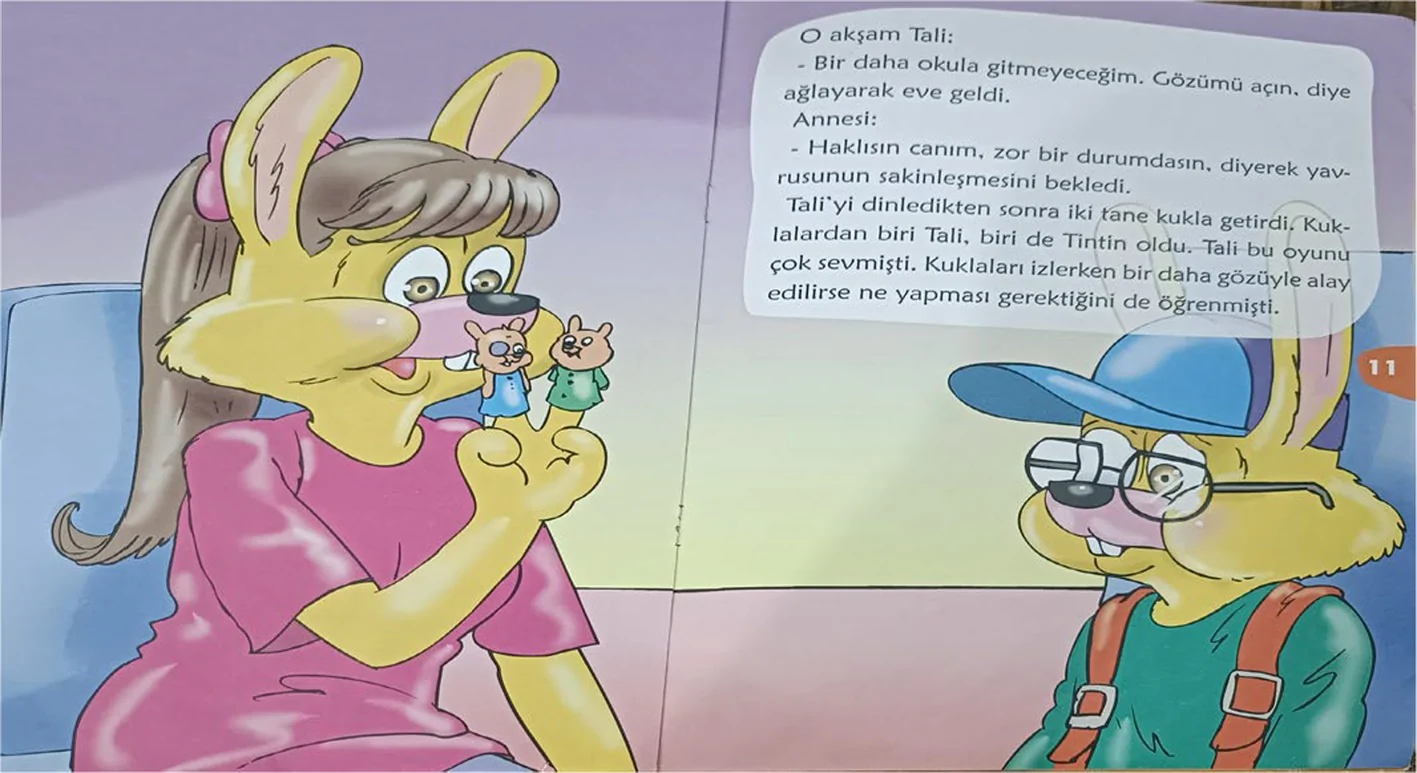
“Tali Copes with Bullying” book (Reproduced with permission from Işıkoğlu, 2018).

The central problem in the book arises from a physical necessity that leads the main character, Tali, to be subjected to peer bullying. Instead of giving advice, the mother’s use of role-playing through puppets and offering strategic support allows the child to protect her self-esteem and develop one possible coping strategy, namely ignoring certain bullying behaviors within the context of the story. The present analysis does not assume that avoidance is appropriate in every bullying situation but interprets it as the specific educational message promoted by this narrative.

Book 6: In the book “Being a Baby Octopus is Very Hard” we see a problem structured around the child’s process of developing self-esteem and accepting their physical characteristics on an individual/internal level. Childhood is a critical period for the formation of self-perception and the development of body awareness. The family’s realistic and heroic problem-solving approach is noteworthy. The family’s role in the process is presented in a supportive manner. In the book, it is seen that the parent strengthens the child’s self-esteem through acceptance, verbal affirmation, and encouragement. In this respect, the book presents family support as an important narrative resource for fostering self-worth. This reflects the values promoted by the story and should not be interpreted as direct evidence of the effectiveness of any single parenting strategy.

In Figure 9, the mother supports the baby octopus in coping with his feelings of self-dislike. “You are very special to us, Nino, you are beautiful just the way you are. It will get easier dressing when you grow a little bigger. You will love being an octopus when you grow and become stronger. How nice it sounded when your mother said that.”

**Figure 9:**
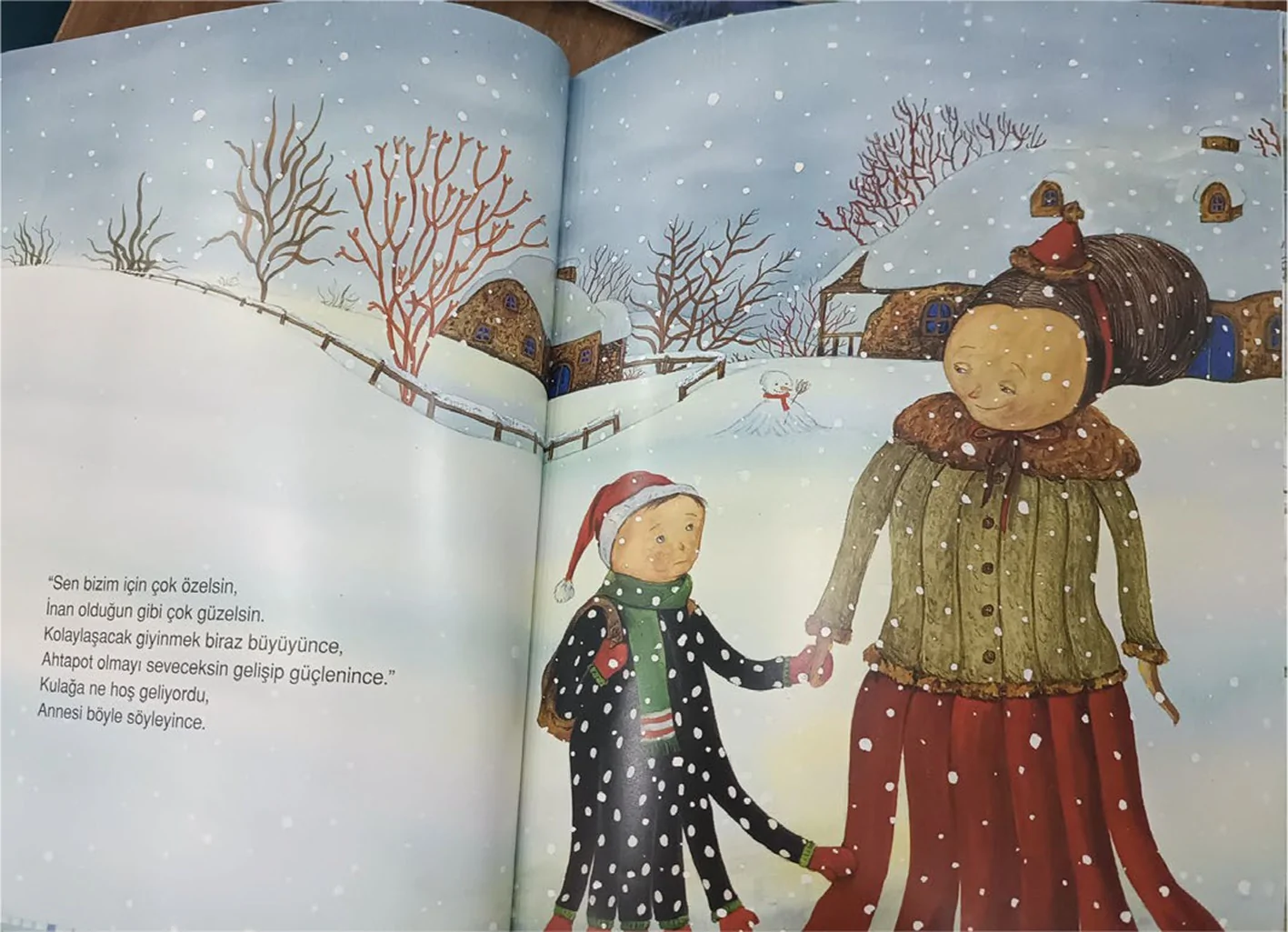
“Being a Baby Octopus is Very Hard” book (Reproduced with permission from Şahinkanat, 2023).

Initially, Nino sees his physical characteristics as an obstacle; however, during a sudden crisis (the collapse of the nest), he sees that these characteristics turn into vital advantages. His parents exhibit a patient and supportive attitude during these moments when Nino feels inadequate, thus demonstrating a model of unconditional acceptance and empowering parenting. The fact that Nino’s self-complaining characteristic serves a social benefit (saving the eggs) can be used as an effective tool to reinforce the reader’s self-worth. The book conveys the message to families that aspects children perceive as weaknesses are actually potentials waiting to be discovered, guiding them through emotional resilience and self-acceptance processes.

Book 7: “The Kissing Hand” addresses the problem of separation anxiety and school phobia in children at an individual/internal level. These attachment-based anxieties are frequently observed during the preschool and primary school transition periods. The book presents a solution approach based on imagination and creativity. Love is transformed into a concrete symbol (a kiss placed in the palm), thus attempting to support the child’s emotional security. The book aims to reduce the child’s anxiety level by making love, an abstract emotion, concrete. The family plays a guiding and supportive role in the problem-solving process. The parent offers a hidden bond that the child can use in coping with anxiety. Thus, the book illustrates a symbolic form of emotional reassurance that the narrative presents as helpful for coping with separation anxiety. The effectiveness of symbolic strategies may vary across children and situations.

In Picture 10, the mother is supporting her baby raccoon in coping with separation anxiety. “I’ll show you. Mother Raccoon took her cub’s left hand, spreading her slender fingers like a fan. Leaning forward, she kissed the very center of Little Raccoon’s palm. Little Raccoon felt his mother’s kiss travel from his hand to his arm, and then to his heart. Even his velvety black mask trembled with a special warmth.”

The book deals with separation anxiety and school adjustment problems. The character of Mother Raccoon is described as a patient, empathetic, and creative guide in this process. The mother particularly concretizes anxiety, an abstract and complex emotion, with the metaphor of “a kiss placed on the palm.” It stands out as a powerful example of how families can provide effective support to their children during times of crisis.

Book 8: The book, “İnci Doesn’t Go with Strangers” addresses the social/security dimension of the problem of stranger danger and the associated risk of rule violations. It is crucial for children to develop safety awareness at an early age and to be able to distinguish risky situations. The book offers a logical and analytical solution to the problem; it shows that the problem is resolved when the child applies the safety rules they have learned beforehand in the appropriate context. The family plays a guiding role in the problem-solving process; it is seen that parents raise the child’s awareness of potential risks by providing preventive education. At the same time, it is seen that they reinforce the child’s correct behavior, supporting their safe decision-making processes. In this respect, the book emphasizes the importance of family guidance in instilling safety-based behaviors in children (see Figure 10).

**Figure 10:**
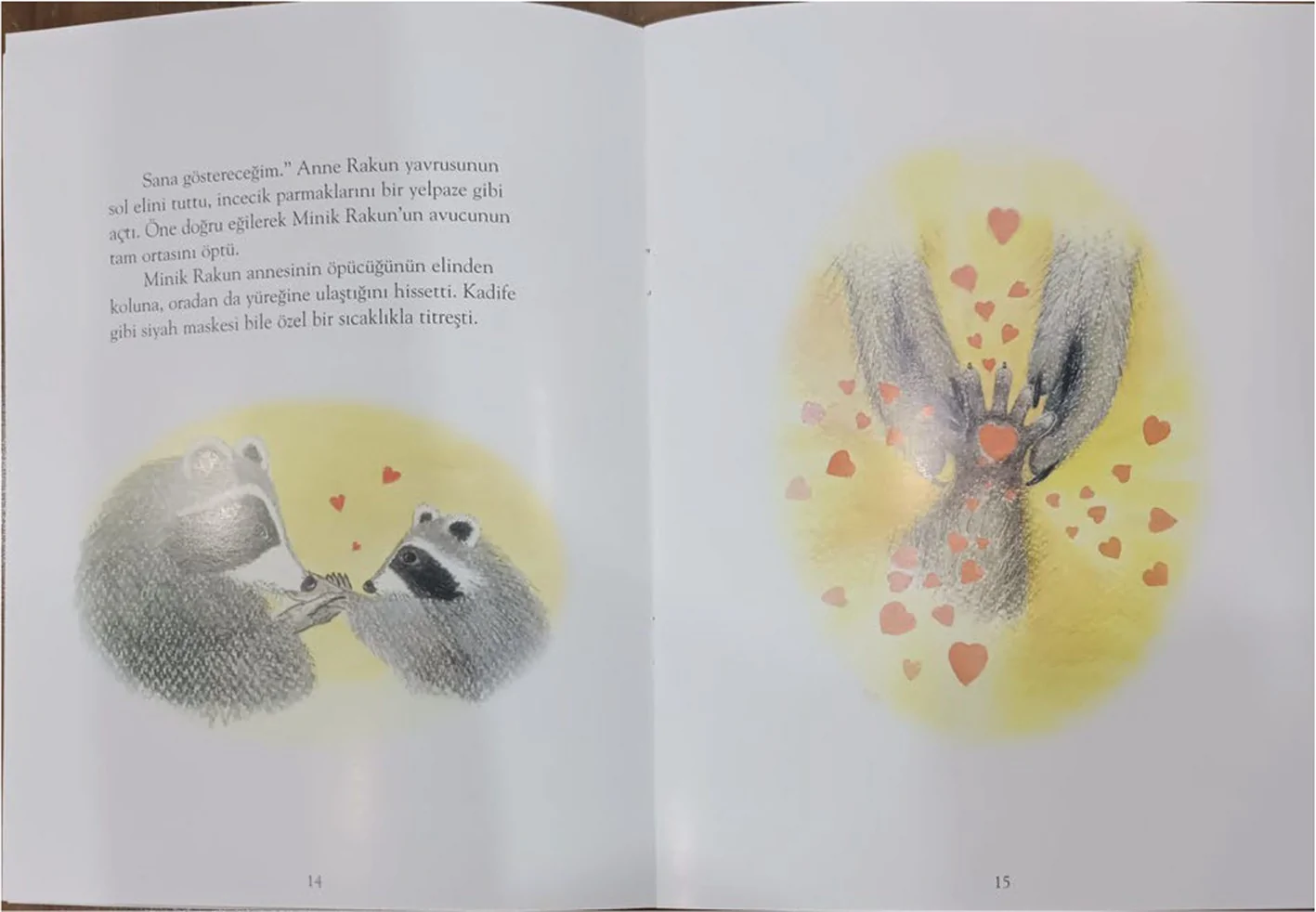
“The Kissing Hand” book (Reproduced with permission from Penn, 2013).

In Figure 11, we see the safety training that İnci’s mother gave her and her application of it. “İnci began to think. She would love to see the kittens. She might even be able to adopt one. But would her mother allow it? Then she suddenly felt warm, then a chill. Because she remembered what her mother always said: Never go with a stranger. No matter how friendly he is or what he promises you. İnci thought, ‘This man might be a good man, but I don’t know him at all. So this man is a stranger.’”

**Figure 11:**
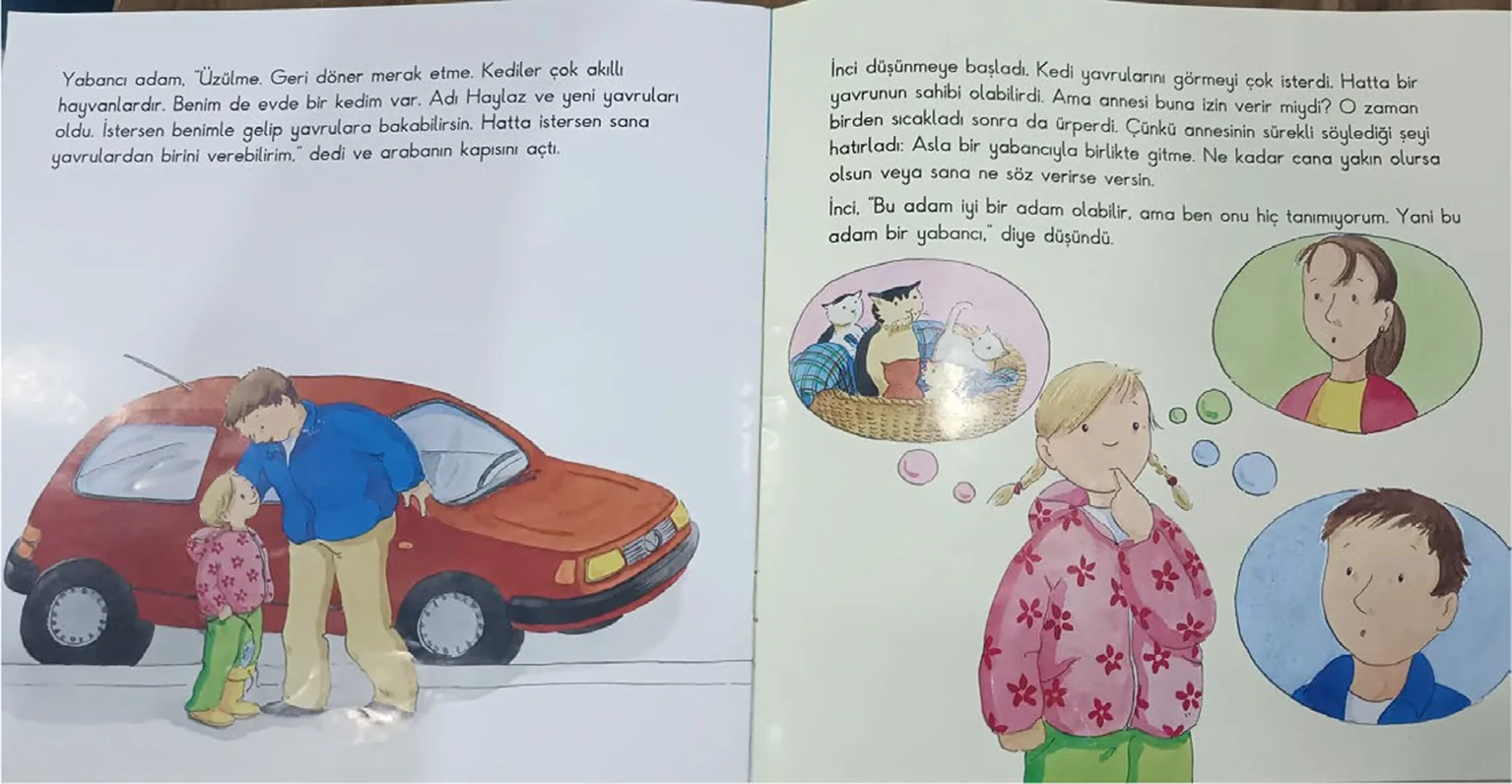
“İnci Does not Go with Strangers” book (Wagenhoff, 2019). “Jule geht nicht mit Fremden mit” book (Reproduced with permission from Wagenhoff, 2009).

The central problem in the book is İnci’s unauthorized outing and exposure to a safety risk by a stranger adult. İnci’s ability to say no to the tempting offer (the promise of a kitten), by heeding the rule she learned from her mother, illustrates how the narrative portrays prior parental safety guidance as supporting the child’s decision-making in a potentially risky situation. This representation promotes precautionary behavior as an educational value; however, it should not be interpreted as suggesting that all interactions with unfamiliar adults should be understood in simplistic terms. Rather, the story reflects one approach to discussing personal safety, trust, and help-seeking with young children. We see that parents instill in their children a encourage children to exercise caution in unfamiliar situations while relying on trusted adults when necessary. The interpretation presented here reflects the educational message of the narrative and acknowledges that children’s understanding of safety and trust develops within broader developmental and social contexts.

Book 9: “Which of Us Do You Love Most?” This book addresses problems such as sibling rivalry and the perception of equality within the family. Children seek to understand their place within the family, and emotional tensions can arise during the process of sharing parental love. The book highlights how conflict is resolved and an appropriate solution approach is idealized through responses emphasizing that parents offer equal and unconditional love to all children. In this context, the work aims to make children feel valued and secure, and provides the reader with an emotional framework. The family plays a supportive role in the problem-solving process. The book shows that parents emphasize the uniqueness of each child, strengthening their individual values rather than comparing them to each other. The work portrays unconditional acceptance as an educational value intended to reduce sibling rivalry within the narrative. Alternative family situations may require different approaches depending on children’s individual needs and family dynamics.

In Figure 12, the mother supports the baby raccoon in coping with feelings of sibling rivalry and the inability to share love. “I know, because your father told me so,” *said their mother.* “I’ll never forget, when he saw you the night you were born, he said: ‘These cubs are the most magnificent bears I’ve ever seen. These cubs are the most magnificent bears ever seen!’”

**Figure 12:**
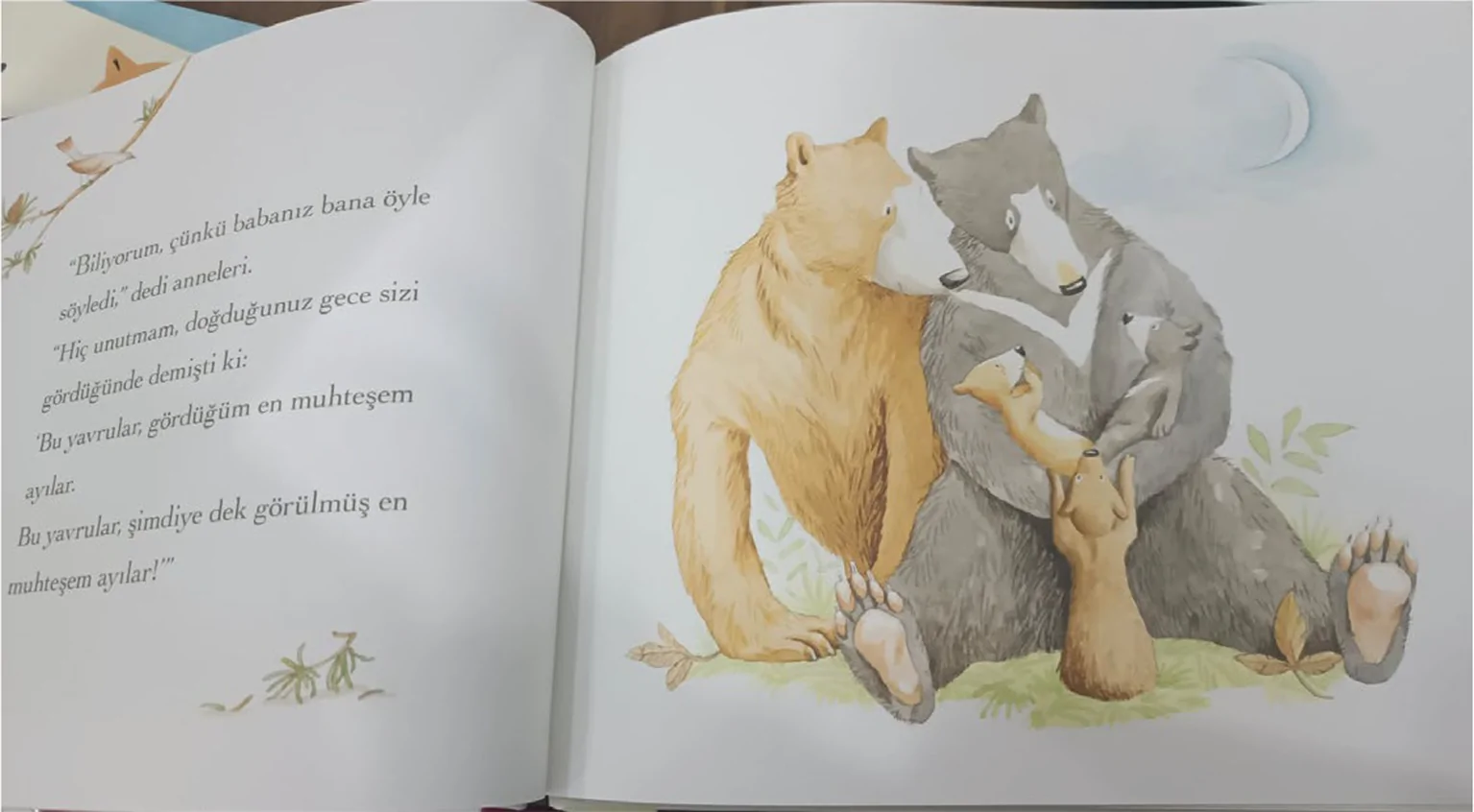
“Which of Us Do You Love Most?” book (Reproduced with permission from McBratney, 2019).

The book shows that the central problem is a feeling of inadequacy stemming from the individual differences of the three different bear cubs. The parents demonstrate an inclusive approach, emphasizing the uniqueness of each cub and offering love not as a divided part, but as a complete whole, recreated for each one. The book conveys the message that parental emotional sharing and honest communication play a critical role in overcoming sibling rivalry and building self-confidence in children. By emphasizing that individual differences within the family are not deficiencies but rather richness that makes each child special, the work highlights the importance of a safe family environment.

Book 10: The book, “Bad Words Smell Bad” is structured around the problem of misunderstanding and communication breakdown. The solution to the problem is based on a logical foundation; misunderstandings are resolved by delving into the root of the problem and questioning the expressions used. This work aims to make children understand the importance of the correct and conscious use of language and to develop critical thinking skills in communication processes. The family plays a problem-solving role in the story; parents manage the resulting confusion and demonstrate a model of correct communication methods. In this respect, the work illustrates one narrative representation of how family dialogue may support children’s understanding of language and communication. This interpretation concerns the educational message conveyed by the story rather than an empirically established parenting practice.

In Figure 13, the mother hedgehog helps the others find the correct information. “Mom, said the baby hedgehog while eating his cake, “what is a bad word? How do I know if a word is bad?” “Oh darling, it’s very easy,” *replied his mother.* “Bad words smell bad.” “Like your burnt cake?” *asked the curious baby hedgehog. The mother hedgehog replied,* “Wash your hands before you eat, you know-it-all!”

**Figure 13:**
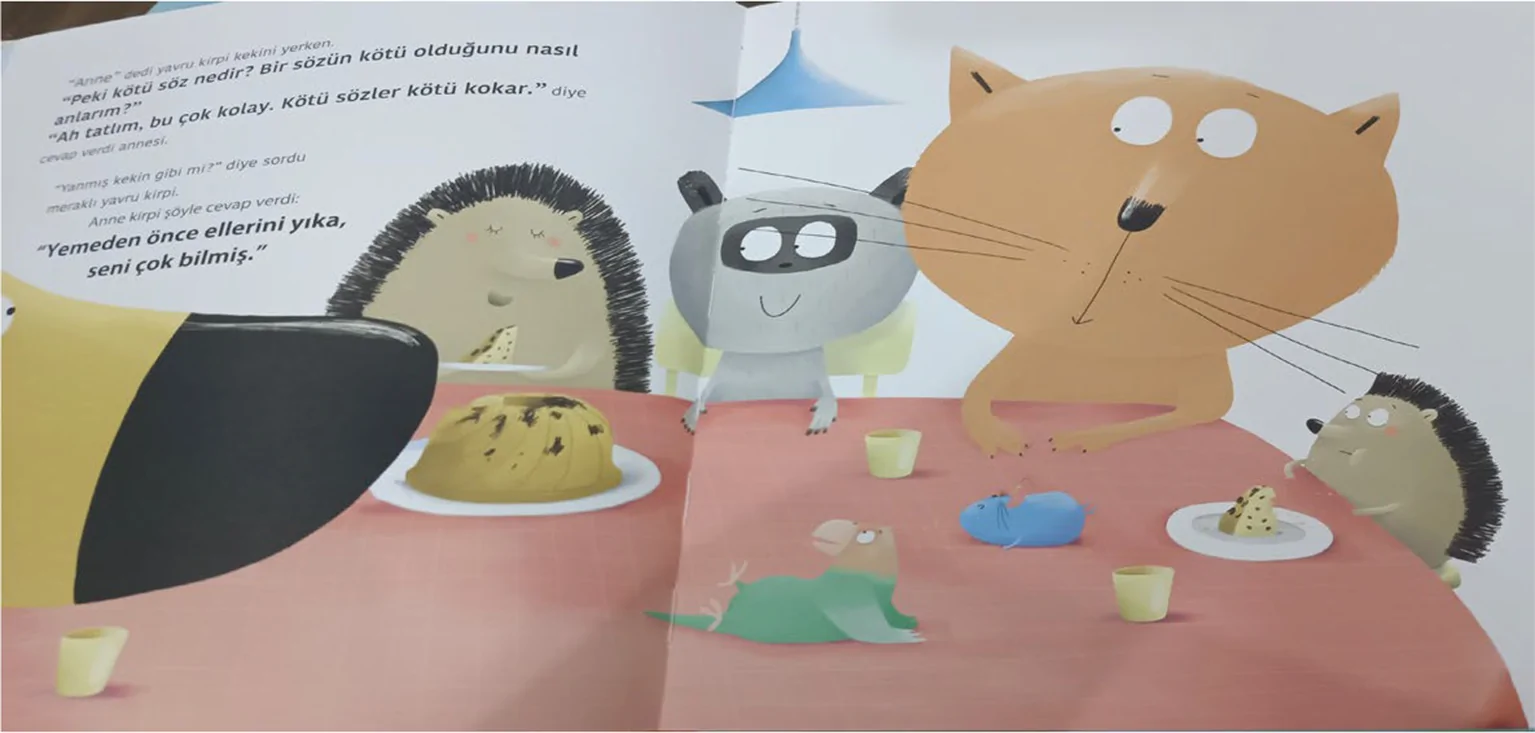
“Bad words smell bad” book (Reproduced with permission from Scarpa, 2020).

The book’s central problem is framed around a misunderstanding (an innocent exclamation being mistaken for a bad word). The Mother Hedgehog character reflects a research-oriented and guiding parent model by investigating the source of the words heard, rather than immediately judging them. The Mother Hedgehog’s concretization of bad words with unpleasant smells that disturb the environment reveals that abstract moral concepts are made understandable. In the book, parents are both encouraged to question the context and intention of words with their children and given the opportunity to turn these perceptions into learning opportunities.

The approaches parents use to help their children solve problems in the 10 illustrated children’s books examined in this research were grouped under four main categories. The first category, “metaphorical concretization and cognitive guidance,” encompasses explaining abstract or complex concepts such as love, belonging, security, property boundaries, and moral values in a way that is appropriate to children’s cognitive levels. This approach is particularly evident in the book “Unshared Pens,” where the act of asking permission is explained using the metaphor of a “key,” in “The Kissing Hand,” where maternal love and attachment are concretized with the symbol of a “kiss placed in the palm,” and in “Bad Words Smell Bad,” where the impact of bad words is explained through the metaphor of a “bad smell.” Similarly, in “İnci Doesn’t Go With Strangers,” the safety training and rules parents provide beforehand regarding interaction with strangers stand out as an example of cognitive guidance that enables the child to make the right decision in a risky situation. These practices contribute to children’s better understanding of abstract rules and complex social situations and their ability to make appropriate decisions.

The second category, “play, role-playing, and creating a safe rehearsal space”, refers to parents enabling children to experience problem situations in a safe environment through gamification, puppet use, dramatization, and interactive activities. This approach is particularly evident in “Tali Copes with Bullying”, where the mother dramatizes solutions to peer bullying through puppets, and in “The Badger Who Couldn’t Sleep”, where the child’s participation in fulfilling their responsibilities is facilitated through play and interaction. Thus, children have the opportunity to experience and apply solution strategies for potential problems in a safe environment.

The third category, “democratic participation and joint decision-making”, encompasses practices that support children’s active participation in family decision-making processes, their expression of opinions, and their making choices. This approach is most clearly seen in “Democracy at Home”, where family members vote on how to use a limited budget, enabling children to actively participate in the decision-making process. Similarly, in “The Badger Who Couldn’t Sleep”, the parent’s granting of choices to the child and their involvement in the solution process reflects a democratic parenting approach. Through these practices, children’s ability to take responsibility, develop autonomy, and think critically is supported.

The fourth category, “emotional validation and unconditional support”, emerged as the most frequently encountered parenting approach in the works examined. In this context, “Will You Still Love Me?” shows the child’s anxiety about not being loved being alleviated through an emphasis on unconditional love; “Unshared Pens” shows sibling conflict being resolved through the expression of feelings and empathy; “Tali Copes with Bullying” shows the mother primarily accepting the child’s feelings; “Being a Baby Octopus is Very Hard” shows parents supporting the child’s self-esteem; “The Kissing Hand” shows separation anxiety being reduced through emotional security; and “Which of Us Do You Love Most?” shows sibling rivalry being addressed through an approach of unconditional love and acceptance. Through this approach, parents create a safe emotional environment by acknowledging children’s fears, anxieties, feelings of inadequacy, and issues related to belonging; thus contributing to the development of children’s self-confidence, emotional resilience, and problem-solving skills.

The solutions developed by parents to the problems their children face, as examined in the works within the scope of the research, were divided into four categories. The first category involved the metaphorical concretization method and cognitive guidance, where complex and abstract concepts such as belonging, love, or property boundaries were made concrete by likening them to objective symbols such as “kiss in the palm” or “a key to asking permission,” and reduced to a level that the child could cognitively understand. The second category involved addressing problems through role-playing, gamification, the use of puppets, or fictional dramatizations, transforming social crises (such as peer bullying) into a safe rehearsal. The third category involved a democratic participation model in problem-solving, allowing children to vote and express their opinions in family decision-making processes, a structural approach that supports children’s personality development. Finally, it was found that parents established a safe environment focused on unconditional love by accepting the child’s anxieties and fears through emotional validation and trust.

## Results and discussion

The family is the most important institution determining a child’s adaptation to their social environment, their ability to communicate healthily with people, and the level of these relationships. Parents’ behavior towards their children is considered a primary resource that helps children understand their environment and give the most appropriate responses in their interactions with people during the socialization process (Erdoğan and Uçukoğlu, 2011). This research aims to determine how and in what ways the parents of the characters in illustrated children’s books help the characters solve the problems in the story. In this context, when the illustrations and text sections of the 10 illustrated children’s books examined were analyzed, it was determined that in all books, one or both parents were involved in the problem-solving process and guided the characters in coping with the problem. In the 10 works examined, the parents: concretized abstract concepts metaphorically in the problem solving process; transformed social crises into safe rehearsals through play; exhibited participatory attitudes that encouraged democratic participation in decision-making processes; and created an unshakeable environment of trust with the emotional approval and reassurance strategies they offered. These narrative representations position parents as supportive guides rather than authority figures and portray family interactions as central elements of children’s problem-solving experiences within the selected picturebooks. Another noteworthy finding concerns the range of family representations within the selected corpus. Although both mothers and fathers appear across the books, their narrative functions are not distributed equally. Mothers are more frequently portrayed as providing emotional reassurance, empathy, and daily caregiving, whereas fathers are more commonly represented as facilitating routines, encouraging participation, or supporting practical problem solving. Nevertheless, both parents are consistently depicted as supportive figures, and no substantial differences in authority or decision-making power were identified within the selected books. The four narrative approaches represent problem solving as a collaborative family process and portray parents as guides rather than rule-makers. Rather than demonstrating actual developmental outcomes, the present findings illustrate how these pedagogical roles are constructed within the narratives of the selected picturebooks. These four narrative approaches portray parents as guides rather than rule-makers and represent problem solving as a collaborative family process within the selected picturebooks. The present study does not demonstrate that these representations improve children’s problem-solving skills; rather, it illustrates how such pedagogical roles are constructed in the narratives. One of the most important findings is the widespread use of emotional coaching strategies. Parents tend to recognize, accept, and validate their children’s negative emotions, such as fear, anxiety, and frustration, before offering solutions. This representation is consistent with previous theoretical and empirical literature suggesting that emotional coaching is associated with children’s emotion regulation, psychological resilience, and psychosocial adjustment. However, because the present study analyzes literary representations rather than children’s developmental outcomes, these findings should be interpreted as narrative portrayals rather than evidence of actual effects on child development. It also supports the family emotion socialization framework proposed by Morris et al. (2007), which suggests that children learn to understand and regulate emotions through observation, communication, and parental responses. In the analyzed books, we see that parents listen to their children’s emotions without suppressing them and transform their emotional experiences into learning opportunities. This finding is particularly important because emotional regulation is seen as a crucial tool between family processes and children’s adjustment. Parents who exhibit a democratic/supportive attitude have more positive communication with their children (Karadağ, 2023). In the works we examined, we see that the communication language of the parents is positive. Within the narratives, this communication style is presented as facilitating the characters’ engagement with problem-solving situations. The study does not assess whether such representations produce similar outcomes among child readers.

It can be said that family demographic characteristics influence attitudes towards the child and the child’s development, as well as the child’s problem-solving skills (Özyürek, 2018). Our findings indicate that the selected picturebooks consistently represent supportive family approaches as being associated with children’s confidence, well-being, and problem-solving efforts. These associations are characteristics of the narratives themselves and should not be interpreted as evidence of actual developmental effects. The books examined by the researcher revealed that families had positive relationships with their children and did not experience conflict in these relationships. Furthermore, it was observed that adults did not serve as role models for children and presented problematic situations that supported problem-solving (Altun et al., 2001; Fox and Schirrmacher, 2014). In the works examined, the characters produce solutions to their problems with the support of their parents, establish strong communication bonds within the family, and positively influence personality development. Illustrated storybooks, prepared in a way that is appropriate to children’s own world and language, serve as an important learning resource for them, and also act as a tool for raising future adults to be qualified and well-equipped (Adlığ, 2023). Illustrated storybooks are effective in supporting children’s problem-solving skills (Balaban, 2018; Begde, 2015; Matemba et al., 2014; Toran, 2020; Tümbul, 2023). The narrative patterns identified in the present study are compatible with these previous findings, although no direct conclusions regarding children’s developmental outcomes can be drawn from the present document analysis.

Problem focused children’s books enhance children’s real-life experiences, improving their ability to cope with challenges (Lukens et al., 2013). Picture books can be used as an effective tool in resolving challenging situations and behaviors (Harper, 2016). Considering the results, the role of families in children’s problem-solving process is significant. Children’s books play a major role in reflecting this reality to children. Encountering different family approaches to solving problems in the books a child reads allows them to prepare for life and discover new experiences.

Research findings indicate that illustrated children’s books portray parents as active guides in children’s problem-solving processes. These findings concern the representational patterns identified in the selected books and should not be interpreted as evidence that exposure to such books directly improves children’s socio-emotional development. Establishing such causal relationships would require experimental, longitudinal, or reader-response studies involving children and families. The works examined show that parents support children in developing solutions by acknowledging their emotions in the face of fear, anxiety, and social conflicts. This aligns with parental emotion socialization theory and parallels current studies demonstrating that emotion coaching strengthens children’s emotion regulation, psychological adjustment, and resilience skills (Guo et al., 2024; Azevedo et al., 2025). Similarly, it is noted that children’s emotion regulation skills play a mediating role in the relationship between intrafamily interactions and psychosocial adjustment (Lin et al., 2024). Furthermore, the concretization of abstract concepts such as love, trust, and belonging through metaphors is highlighted as an effective pedagogical strategy for developing children’s emotional awareness (Aldossary, 2026). In addition, gamification, role-playing, and democratic participation practices have been shown to support children’s social–emotional learning and problem-solving skills (Parker and Cronin, 2025; Spinrad and Eisenberg, 2023). These findings indicate that within the 10 picturebooks examined, family members are predominantly represented as supportive guides who facilitate children’s emotional and social problem-solving. These conclusions are intentionally limited to the selected corpus and should not be interpreted as representing contemporary picturebooks in general or children’s literature across different languages and cultures.

Across the 10 picturebooks, family members consistently adopted supportive rather than punitive approaches to problem solving. However, the form of support differed across books. While some stories emphasized emotional reassurance and empathy, others highlighted cognitive guidance through questioning or encouraged children to actively participate in finding solutions. These variations suggest that contemporary picturebooks present multiple pedagogical models of family involvement rather than a single dominant approach. An equally important observation is the absence of ineffective, neglectful, or harmful parental responses across the corpus. All parental interventions are presented as constructive, supportive, and ultimately successful in helping children cope with difficulties. Consequently, the selected books construct a predominantly idealized image of family life in which conflicts are resolved through empathy, communication, and guidance. While this positive representation may serve educational purposes, it also limits the diversity of parenting practices represented within the corpus.The findings may also be interpreted within broader theoretical perspectives on children’s learning and development. The frequent representation of parents providing guidance, encouragement, and gradual support is consistent with Vygotsky’s concept of scaffolding, which emphasizes that children acquire higher order cognitive and problem-solving skills through supportive interactions with more knowledgeable others (Vygotsky and Cole, 1978). Likewise, the depiction of parents as role models who demonstrate constructive coping strategies aligns with social Learning Theory, suggesting that children learn social behaviors by observing and imitating significant adults (Bandura and Walters, 1977). Furthermore, the prominence of emotional validation and supportive communication across the picturebooks reflects key principles of emotion socialization theory, which proposes that parents’ responses to children’s emotions contribute to the development of emotional competence and adaptive coping (Eisenberg et al., 1998; Morris et al., 2007). It should be noted, however, that the present findings refer to the representation of these practices in illustrated children’s books rather than their direct effects on children’s development.

Based on this study, the following suggestions can be made: It should not be forgotten that children’s literature works need to be carefully designed and written. The works created should be designed in a way that the family serves as a positive role model, and authors should avoid negative connotations. However, care should be taken not to detach from reality when portraying the family structure. In this context, the author should create the narrative considering the developmental characteristics of the age group they are addressing. Increasing the number of children’s literature books that address the family and its importance would be beneficial. Highlighting the influence of family members on a child’s personality development and their experience in problem solving, and emphasizing the importance of the family, is crucial. Experimental studies can be designed using children’s literature works that deal with the family theme to determine the extent to which these books influence children’s views of family. Research can be conducted comparing the approaches presented in books to problem solving with children’s approaches to problem solving.

## Limitations

This study has several limitations that should be acknowledged. First, the findings are based on a relatively small purposive sample of 10 picturebooks; therefore, the results should not be generalized beyond the selected corpus. Second, because one of the inclusion criteria required books to contain family-supported problem-solving situations, the corpus naturally emphasized supportive parental representations while excluding narratives depicting ineffective or harmful family interactions. Third, the corpus was limited to books available within a particular linguistic and publishing context, which may not fully represent picturebooks published in other languages or cultural settings. Fourth, the study relied exclusively on document analysis and did not include reader-response data from children, parents, or teachers. Consequently, no conclusions can be drawn regarding how children actually interpret these narratives or whether the represented parenting practices influence children’s attitudes or behaviors. Fifth, although systematic coding procedures were followed, qualitative interpretation inevitably reflects the analytical perspective of the researchers. Finally, while intercoder agreement procedures were implemented, more detailed reporting of coding consistency would further strengthen methodological transparency. Accordingly, the findings should be interpreted as representations identified within the selected books rather than evidence regarding the developmental effectiveness or prevalence of these parenting approaches in children’s literature more broadly.

## Data Availability

The original contributions presented in the study are included in the article/Supplementary material, further inquiries can be directed to the corresponding author.
