# Supplementary material for: Parental guidance and problem solving in illustrated children’s books: the representation of the family as a pedagogical actor
Source: Front Psychol. 2026 Sep 30;17:1915321. doi: 10.3389/fpsyg.2026.1915321 (PMC13662878; doi:10.3389/fpsyg.2026.1915321)
Supplement: Supplementary file 1 [file Table_1.DOCX]

# **Supplementary Material**

## **Appendix S1. Corpus Description**

The study corpus consisted of ten illustrated children’s books published between 2013 and 2024. The books were selected according to the inclusion criteria described in the manuscript. All books contained a child protagonist, a problem-solving episode, and the active involvement of at least one family member in the problem-solving process. The corpus included both human and animal protagonists in order to reflect the diversity of contemporary picturebooks while maintaining comparable narrative structures.

### Table S1. Characteristics of the Picturebook Corpus

| Book ID | Title | Publisher | Publication Year | Character Type | Family Representation |
| --- | --- | --- | --- | --- | --- |
| B1 | *Will You Still Love Me?* | Meav | 2021 | Animal | Family members participate in resolving the child’s problem. |
| B2 | *Democracy at Home* | Eğiten Kitap | 2018 | Animal | Family members participate in resolving the child’s problem. |
| B3 | *Unshared Pens* | Cezve Children | 2021 | Human | Family members participate in resolving the child’s problem. |
| B4 | *The Badger Who Couldn’t Sleep* | İş Kültür Publishing | 2021 | Animal | Family members participate in resolving the child’s problem. |
| B5 | *Tali Copes with Bullying* | Nesil Children’s Publications | 2018 | Animal | Family members participate in resolving the child’s problem. |
| B6 | *Being a Baby Octopus is Very Hard* | Yapı Kredi Publications (YKY) | 2023 | Animal | Family members participate in resolving the child’s problem. |
| B7 | *The Kissing Hand* | Butik Publishing | 2013 | Animal | Family members participate in resolving the child’s problem. |
| B8 | *İnci Doesn’t Go with Strangers* | İş Kültür Publishing | 2018 | Human | Family members participate in resolving the child’s problem. |
| B9 | *Which of Us Do You Love Most?* | Uçanbalık Publishing | 2024 | Animal | Family members participate in resolving the child’s problem. |
| B10 | *Bad Words Smell Bad* | Nar Publishing | 2020 | Animal | Family members participate in resolving the child’s problem. |

## **Book Information Form**

For each picturebook, the following descriptive information was recorded before coding:

- Book identification code
- Title
- Publisher
- Publication year
- Character type (human/animal)
- Presence of a child protagonist
- Family member(s) involved
- Type of problem presented
- Problem-solving episode(s)
- Type(s) of parental guidance represented
- Narrative outcome
- Verbal evidence
- Visual evidence
- Final coding category (or categories)

The Book Information Form was completed for each of the ten picturebooks prior to thematic coding and served as the basis for the subsequent content analysis.

## **Appendix S2. Synthesis Table of the Coding Results**

| Book ID | Picturebook Title | Type of Problem | Family Member Involved | Child Character’s Response | Parental Strategy | Textual Indicator | Visual Indicator | Assigned Code & Category | Ambiguities / Alternative Interpretations |
| --- | --- | --- | --- | --- | --- | --- | --- | --- | --- |
| B1 | *Will You Still Love Me?* | Fear of losing parental love / emotional insecurity | Mother | Expresses concerns and seeks reassurance | Emotional Coaching | Mother verbally reassures the child that parental love is unconditional. | Warm facial expressions, physical closeness, affectionate gestures. | Emotional Coaching | The reassurance may also be interpreted as promoting emotional security. |
| B2 | *Democracy at Home* | Family decision-making | Mother and Father | Shares opinions and participates in decisions | Democratic Participation | Parents invite the child to express ideas before making a decision. | Family members are positioned equally around the table, indicating participation. | Democratic Participation | Some interactions may also reflect cognitive guidance. |
| B3 | *Unshared Pens* | Sharing and peer conflict | Mother | Reflects on own behaviour and shares materials | Cognitive Guidance | Parent explains the importance of sharing and asks reflective questions. | Child and parent maintain eye contact while discussing the situation. | Cognitive Guidance | The strategy also contains elements of moral modeling. |
| B4 | *The Badger Who Couldn’t Sleep* | Fear / difficulty sleeping | Mother | Follows the suggested coping strategy and calms down | Emotional Coaching | Parent comforts and reassures the child before bedtime. | Soft colours, close body posture, calm facial expressions. | Emotional Coaching | Visual atmosphere reinforces emotional regulation. |
| B5 | *Tali Copes with Bullying* | Bullying | Mother | Reports the problem and follows guidance | Cognitive Guidance | Parent discusses alternative ways to respond to bullying. | Supportive body posture and attentive listening are emphasised. | Cognitive Guidance | Could also be interpreted as emotional coaching. |
| B6 | *Being a Baby Octopus is Very Hard* | Difficulty learning everyday skills | Mother | Tries again after observing the parent | Modeling | Parent demonstrates how to perform the task step by step. | Sequential illustrations show imitation of the demonstrated behaviour. | Modeling | Includes elements of safe rehearsal. |
| B7 | *The Kissing Hand* | Separation anxiety | Mother | Gains confidence and attends school | Emotional Coaching | Parent provides emotional reassurance through a symbolic gesture. | The kissing hand gesture and affectionate embrace reinforce the message visually. | Emotional Coaching | Symbolic gesture extends the verbal narrative. |
| B8 | *İnci Doesn’t Go with Strangers* | Personal safety | Mother | Learns safety rules and applies them | Cognitive Guidance | Parent explains why strangers should be avoided. | Warning gestures, protective positioning, and eye contact support the verbal message. | Cognitive Guidance | Some scenes also demonstrate external regulation. |
| B9 | *Which of Us Do You Love Most?* | Sibling jealousy | Mother | Understands parental explanation and accepts the situation | Emotional Coaching | Parent explains that love for each child is equal. | Parent embraces both children equally, reinforcing the verbal explanation. | Emotional Coaching | Visual evidence strengthens the narrative message. |
| B10 | *Bad Words Smell Bad* | Inappropriate language use | Mother | Recognises inappropriate behaviour and changes it | Modeling / Cognitive Guidance | Parent explains respectful communication and models appropriate language. | Facial expressions and behavioural modelling illustrate appropriate communication. | Modeling; Cognitive Guidance | Both categories are equally applicable depending on interpretive emphasis. |

# **Supplementary File 1. Record of Category Development**

| **Stage** | **Description** |
| --- | --- |
| Initial Framework | Initial codes generated from literature and research questions |
| First Coding | Books independently coded |
| Code Revision | Similar codes merged |
| Category Formation | Codes grouped into higher-order categories |
| Final Themes | Four overarching themes established after consensus |

# **Supplementary File 2. Coding Checklist(Tali Copes with Bullying)**

**Each book was evaluated according to the following checklist.**

| **Criterion** | **Yes** | **No** |
| --- | --- | --- |
| Family member participates in solving the problem | x | □ |
| Child experiences a clearly defined problem | x | □ |
| Parent uses an identifiable strategy | x | □ |
| Strategy supported by textual evidence | x | □ |
| Strategy supported by visual evidence | x | □ |
| Child's response identifiable | x | □ |
| Final code assigned | x | □ |
| Theme assigned | x | □ |
